# A study on the prevalence and related factors of frailty and pre-frailty in the older population with hypertension in China: A national cross-sectional study

**DOI:** 10.3389/fcvm.2022.1057361

**Published:** 2023-01-12

**Authors:** Xue-zhai Zeng, Na Jia, Ling-bing Meng, Jing Shi, Ying-ying Li, Jia-bin Hu, Xing Hu, Hui Li, Hong-xuan Xu, Jian-yi Li, Xin Qi, Hua Wang, Qiu-xia Zhang, Juan Li, De-ping Liu

**Affiliations:** ^1^Department of Cardiology, Beijing Hospital, National Center of Gerontology, Institute of Geriatric Medicine, Chinese Academy of Medical Sciences, Beijing, China; ^2^Department of Geriatrics, Beijing Hospital, National Center of Gerontology, Institute of Geriatric Medicine, Chinese Academy of Medical Sciences, Beijing, China; ^3^Health Service Department of the Guard Bureau of the Joint Staff Department, Beijing, China; ^4^China Research Center on Aging, Beijing, China; ^5^Institute of Psychology, Chinese Academy of Sciences, Beijing, China

**Keywords:** older adults, hypertension, frailty, pre-frailty, related factors, prevalence

## Abstract

**Objective:**

To explore the prevalence and factors associated with frailty and pre-frailty in elderly Chinese patients with hypertension.

**Background:**

In China, there have been few national studies into the prevalence and factors associated with frailty and pre-frailty in elderly patients with hypertension.

**Methods:**

Through the 4th Sample Survey of Aged Population in Urban and Rural China (SSAPUR) in 2015, the situation of hypertension subjects aged 60 years or older in 31 provinces, autonomous regions, and municipalities in mainland China was obtained. And the frailty index was constructed based on 33 potential defects, elderly hypertensive patients are classified as robust, frailty, and pre-frailty.

**Results:**

A total of 76,801 elderly patients with hypertension were enrolled in the study. The age-sex standardized prevalence of frailty and pre-frailty in hypertensive elderly in China was 16.1% (95%CI 15.8–16.3%), 58.1% (95%CI 57.7–58.4%). There were significant geographical differences in the prevalence of frailty and pre-frailty in elderly hypertensive patients. Multinomial logistic regression analysis showed that poor economic status, activities of daily living disability, and comorbid chronic diseases were related to frailty and pre-frailty.

**Conclusion:**

Frailty and pre-frailty are very common in elderly Chinese patients with hypertension and have similar risk factors. Prevention strategies should be developed to stop or delay the onset of frailty by targeting established risk factors in the pre-frailty population of elderly hypertension. It is also crucial to optimize the management of frailty in elderly Chinese patients with hypertension.

## Introduction

At least 50% of older adults suffer from hypertension, and the prevalence of hypertension in the elderly over 80 years is close to 90% (1, 2). Hypertension is the foremost independent risk factor for stroke, myocardial infarction, and even cardiovascular death in older adults (3, 4). Frailty is a non-specific state of progressive decline in the function and reserve of multiple systems, diminished homeostatic regulation of the body, and decreased compensatory capacity for physiological adaptation in response to stress in the elderly (5). Global community of elderly frail prevalence was 4–59%, and the average prevalence was 11% (6). This leads to an increase in negative clinical events (4, 5, 7, 8), also leading to increased long-term care needs and medical costs for older adults (9). A meta-analysis revealed that frail older hypertensive patients had a higher risk of death, hospitalization, and falls than older hypertensive patients who were not frail (8). Studies have shown that frailty is a factor that must be considered in setting target blood pressure levels for elderly patients with hypertension (10–12). European Society of Hypertension, European Geriatric Medicine Society, and the 2019 Chinese Guideline for Hypertensive elderly all clearly emphasize that frailty assessment as a concern in the management of hypertension in the elderly (13, 14).

A nationwide survey of frailty in elderly Chinese hypertensive patients found a 13.8% frailty rate in elderly hypertensive patients through a study of 3,844 elderly hypertensive patients from seven cities in China. However, the sample is not rich in frail individuals, and the selection of rural Beijing and rural Chengdu as representatives of rural northern and southern China limited the generalization of the findings (15). At present, there is no study on the prevalence of frailty and pre-frailty in elderly hypertensive patients in various provinces, municipalities and autonomous regions in mainland China. In our study, we used data from the 4th SSAPUR in 2015; we then assessed the frailty and pre-frailty of elderly hypertensive patients by using the frailty index (FI). As far as we know, this is the largest study of frailty in elderly hypertensive patients in China ever conducted. This study aimed to find the prevalence and factors of frailty and pre-frailty in elderly Chinese patients with hypertension in various provinces, municipalities, and autonomous regions in mainland China and provide a basis for the management of older hypertensive patients with frailty.

## Materials and methods

### Study design and participants

**FIGURE 1:**
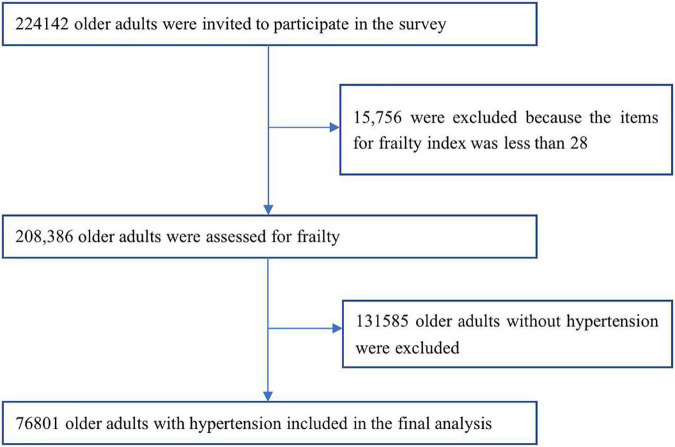
Flowchart of study participants on frailty and pre-frailty prevalence in older adults with hypertension in China.

Content from the 4th SSAPUR, a cross-sectional study of elderly subjects aged 60 years or older from 31 provinces, autonomous regions, and municipalities in mainland China was obtained. The structure and sampling method of SSAPUR have been described in previous studies (16, 17). The number of samples collected by SSAPUR was 224,142, of which 15,756 (7.0%) were excluded because the number of constructed FI items was less than 28. Among the 208,386 older individuals, 76,801 physician-diagnosed elderly patients with hypertension were enrolled in our study (Figure 1). The study protocol was approved by the National Bureau of Statistics (No. [2014] 87) and the ethics committee of Beijing Hospital (2021BJYYEC-294-01). All participants provided written informed consent.

### Demographics

Demographic characteristics: age (60–64, 65–69, 70–74, 75–79, 80–84, and ≥85 years), sex, education (illiterate, primary school, junior secondary school, senior secondary school, tertiary, university or above), marital status (widowed, divorced, unmarried, married), ethnicity (Han, non-Han), residence location (urban, rural), living status (living alone, not living alone), health checkup within 1 year, hospitalized within 1 year, economic status self-assessed based on categories (very rich, rich, adequate, poor, very poor), convenience of medical cost reimbursement (highly convenient, convenient, less convenient, inconvenient, highly inconvenient), activities of daily living (ADL) disability (Inability to do one or more of the following: bathing, dressing, toileting, getting in and out of bed, eating and moving around the room is considered a disability), comorbid chronic diseases (cataract/glaucoma, diabetes, cardiovascular disease, Stomach disease, osteoarthropathy, chronic lung disease, Asthma, Malignant tumor, reproductive system disease), and living in southern or northern China.

### Identification and assignment of health deficit variables for FI

We constructed FI following Searle’s standard procedure (18). The exact construction method has been described in our previous study (16). FI scores greater than or equal to 0.25 are considered frailty, <0.12 are considered robust, and FI 0.12–0.25 are considered pre-frailty.

### Statistical analysis

The prevalence of frailty in older patients with hypertension reported from previous study was 14% (15). We calculated the sample size using the PASS software and applied a design effect of 2.8 to account for the multi-stage cluster sampling design and calculated a sample size of at least 6,804 for our study. therefore, our sample of over 76,000 participants in this study was adequate. SPSS 24.0 software was used for analysis. Missing data were interpolated using Markov Chain Monte Carlo (MCMC) multiple fill method (19). Age-sex standardized prevalence of frailty and pre-frailty among elderly patients with hypertension in China was calculated according to the weights created in our study. We assessed the significance of differences by ANOVA or Student’s *t*-test for continuous variables and by the χ^2^ test for categorical variables. The trend of prevalence by covariables was tested by use of the Cochran–Armitage test. Multinomial regression analysis were used to ascertain the factors associated with frailty and pre-frailty, including comorbidities, ADL disability, southern or northern China, age group, sex, nationality, residence location, education level, marital status, living alone, economic status, medical insurance, the convenience of medical expense reimbursement. *p* < 0.05 was considered statistically significant.

## Results

Among 208,386 elderly participants, the prevalence of frailty was 9.4%, and the prevalence of pre-frailty was 45.8%. The number of hypertensive patients was 76,801 out of 208,386 elderly participants, and the prevalence of hypertension was 36.9%. The prevalence of hypertension was 62.8% in frail elderly, 46.7% in pre-frail elderly, and 21.3% in robust elderly.

The general characteristics and associated factors of the elderly patients with hypertension are shown in Table 1, by grades of frailty. The mean age of hypertensive patients was 70.5 ± 7.8 years (range 60–109 years); 42,966 (55.9%) were women, the mean age of 70.8 ± 7.9 years, 33,835 (44.1%) were men, and mean age of 70.2 ± 7.5 years.

**TABLE 1:** Demographics of the Chinese hypertensive adults aged 60 years or older in 2015, and related factors for frailty, by frailty stage.

	Men	Women		Men				Women			
	**Total (33,835)**	**Total (42,966)**	***p* for difference**	**Robust (10,028)**	**Pre-frailty (19,165)**	**Frailty (4,642)**	***p* for difference**	**Robust (9,845)**	**Pre-frailty (25,451)**	**Frailty (7,670)**	***p* for difference**
Proportion of participants	44.1%	55.9%		29.6%	56.6%	13.7%		22.9%	59.2%	17.9%	
Age (years)	70.2 ± 7.5	70.8 ± 7.9	<0.001	68.3 ± 6.8	70.4 ± 7.5	73.3 ± 8.2	<0.001	68.5 ± 7.0	70.8 ± 7.8	73.7 ± 8.5	<0.001
Age group			<0.001				<0.001				<0.001
60–64	28.4%	27.1%		36.5%	26.8%	17.6%		36.6%	26.5%	17.3%	
65–69	24.6%	23.0%		27.2%	24.6%	19.2%		26.7%	23.0%	18.4%	
70–74	18.7%	18.2%		17.2%	19.4%	18.7%		16.3%	19.0%	17.8%	
75–79	14.7%	15.2%		11.1%	15.5%	19.4%		11.2%	15.6%	18.9%	
80–84	9.0%	10.6%		5.7%	9.2%	15.0%		6.3%	10.6%	15.9%	
≥85	4.6%	5.9%		2.3%	4.5%	10.1%		2.9%	5.3%	11.7%	
Rural residents	43.8%	44.7%	0.019	36.2%	45.9%	51.6%	<0.001	37.3%	45.4%	51.9%	<0.001
Education level			<0.001				<0.001				<0.001
Illiterate	13.6%	43.2%		9.0%	14.5%	19.4%		33.3%	43.4%	55.1%	
Primary school	43.7%	37.0%		40.7%	44.4%	47.6%		40.2%	37.3%	32.0%	
Junior secondary school	26.3%	12.8%		29.8%	25.6%	21.7%		17.1%	12.5%	8.5%	
Senior secondary school	10.5%	5.2%		13.0%	10.0%	7.7%		6.9%	5.1%	3.2%	
Tertiary	3.8%	1.2%		5.1%	3.4%	2.1%		1.7%	1.0%	0.9%	
University or above	2.1%	0.6%		2.4%	2.1%	1.5%		0.8%	0.7%	0.3%	
Ethnicity (Minority)	5.1%	5.1%	0.864	4.0%	5.5%	5.8%	<0.001	4.3%	5.3%	5.6%	<0.001
Marital status			<0.001				<0.001				<0.001
Marital status (married)	81.8%	61.8%		89.6%	79.9%	72.8%		74.1%	61.1%	48.1%	
Widowed	15.2%	37.6%		8.9%	16.4%	23.7%		25.3%	38.2%	51.2%	
Divorced	1.0%	0.5%		0.7%	1.2%	0.9%		0.5%	0.6%	0.5%	
Unmarried	2.0%	0.1%		0.8%	2.5%	2.6%		0.1%	0.1%	0.2%	
Living alone	10.8%	16.7%	<0.001	4.1%	13.0%	16.4%	<0.001	5.6%	18.6%	24.6%	<0.001
Health checkup within 1 year	63.4%	62.0%	<0.001	65.0%	63.6%	59.0%	<0.001	64.3%	62.6%	57.1%	<0.001
Hospitalized within 1 year	30.5%	31.5%	0.004	18.8%	31.7%	51.1%	<0.001	18.4%	31.4%	48.6%	<0.001
Medical insurance (none)	0.9%	0.8%	0.444	0.7%	0.9%	1.0%	0.140	0.9%	0.8%	0.9%	0.523
Convenience of medical expense reimbursement			0.717				<0.001				<0.001
Highly convenient	33.2%	33.2%		36.0%	32.4%	30.3%		35.0%	33.0%	31.2%	
Convenient	43.7%	43.9%		44.2%	43.4%	44.0%		44.7%	43.7%	43.6%	
Less convenient	17.4%	17.4%		15.7%	18.2%	17.8%		16.3%	17.7%	18.1%	
Inconvenient	3.8%	3.7%		2.8%	4.0%	5.5%		2.6%	3.9%	4.7%	
Highly inconvenient	1.9%	1.8%		1.3%	2.0%	2.4%		1.3%	1.7%	2.4%	
Economic status			0.012				<0.001				<0.001
Very rich	1.4%	1.3%		2.1%	1.1%	0.9%		2.3%	1.0%	0.7%	
Rich	16.0%	15.2%		21.7%	14.4%	9.9%		21.6%	14.2%	10.3%	
Adequate	58.8%	59.0%		62.4%	58.6%	52.2%		62.5%	59.6%	52.6%	
Poor	19.8%	20.4%		12.5%	21.7%	27.9%		12.1%	21.2%	28.4%	
Very poor	4.0%	4.1%		1.3%	4.2%	9.1%		1.5%	4.0%	8.0%	
Living in Northern China	26.5%	27.2%	0.021	21.3%	27.0%	35.5%	<0.001	21.8%	27.0%	34.9%	<0.001
Comorbidity (≥1)	76.9%	81.7%	<0.001	42.1%	90.0%	97.9%	<0.001	44.8%	91.1%	98.1%	<0.001
ADL disability	5.1%	6.2%	<0.001	0.1%	1.8%	29.6%	<0.001	0.1%	2.0%	28.0%	<0.001

ADL, activities of daily living.

The distribution of FI in hypertensive elderly was gamma distributed (statistic = 0.093, *P* < 0.001, see Supplementary Figure 1), ranging from 0.00 to 0.70, with a median of 0.16 (0.10). Median FI was 0.15 (0.09) for men, 0.17 (0.10) for women. FI value for women was high (*z* = −26.068, *P* < 0.001).

### Prevalence of frailty and pre-frailty in elderly patients with hypertension

**FIGURE 2:**
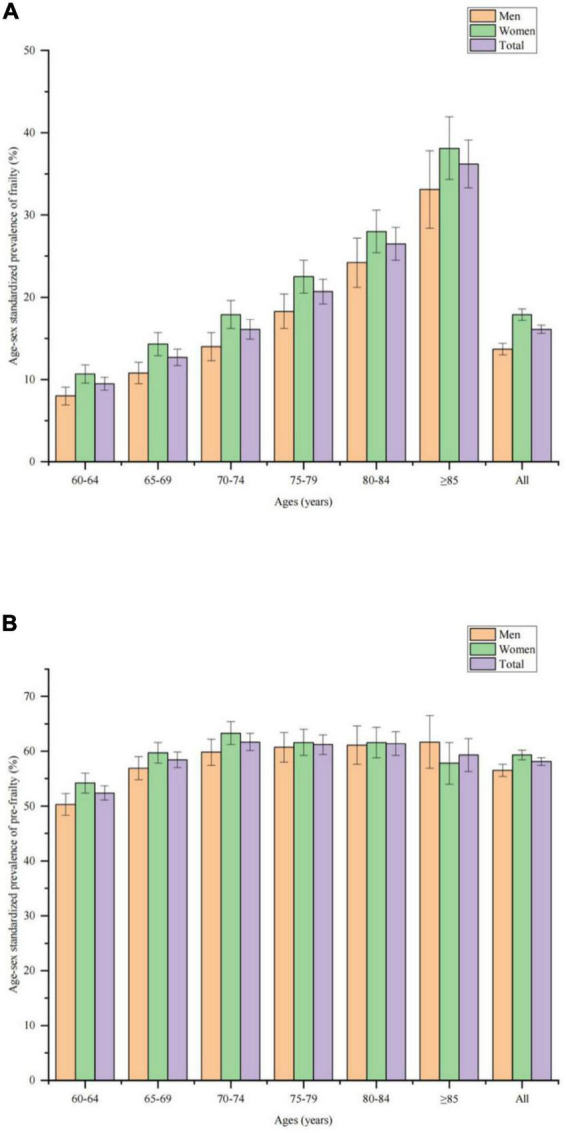
Age-sex standardized prevalence of frailty and pre-frailty in older adults with hypertension. **(A)** Age-sex standardized prevalence of frailty among men, women, and all older adults with hypertension. **(B)** Age-sex standardized prevalence of pre-frailty among men, women, and all older adults with hypertension.

The prevalence of frailty was 16.0% in the elderly with hypertension, higher than in those without hypertension (5.5%; χ^2^ = 6254.922, *P* < 0.001). The prevalence of pre-frailty was 58.1% in the elderly with hypertension, which was higher than in those without hypertension (38.6%; χ^2^ = 7397.020, *P* < 0.001). The age-sex standardized prevalence of frailty and pre-frailty in hypertensive elderly in China was 16.1% (95%CI 15.8–16.3%) and 58.1% (95%CI 57.7–58.4%), respectively. The estimated number of frail elderly hypertensive patients in mainland China in 2015 was 19.0 (18.7–19.3) million, and the number of pre-frail elderly hypertensive patients was 68.6 (68.2–68.9) million. According to the 2020 census, the number of frail and pre-frail elderly hypertensive patients in mainland China was estimated to be 22.5 (22.2–22.9) million and 81.5 (81.0–82.0) million. The age-sex standardized prevalence of frailty [17.9% (17.6–18.3%) vs. 13.7% (13.3–14.0%), *P* < 0.001] and pre-frailty [59.3% (58.8–59.7%) vs. 56.5% (56.0–57.0%, *P* < 0.001)] in older hypertensive patients was higher in women than in men. Age-sex standardized prevalence of frailty in elderly hypertensive patients increased with age, as shown in Figure 2. The prevalence of age-sex standardized frailty [18.5% (18.1–18.9%) vs. 14.0% (13.7–14.4%), *P* < 0.001] and pre-frailty [59.1% (58.5–59.6%) vs. 57.2% (56.7–57.7%), *P* < 0.001] in older hypertensive patients was higher in rural than in urban areas. Frailty and pre-frailty in elderly hypertensive patients were mostly seen in widowed/unmarried, ethnic minorities, living alone, hospitalization in the past year, financial difficulties, inconvenient medical reimbursement, and comorbid chronic diseases, as shown in Table 2.

**TABLE 2:** Prevalence of frailty and pre-frailty among older adults with hypertension in China in 2015.

	Entire population			Men			Women		
	**Prevalence of pre-frail**	**Prevalence of frail**	***P-*value**	**Prevalence of pre-frail**	**Prevalence of frail**	***P-*value**	**Prevalence of pre-frail**	**Prevalence of frail**	***P-*value**
Proportional of participants	58.1%	16.0%		56.6%	13.7%		59.2%	17.9%	
Age group			<0.001			<0.001			<0.001
60–64	55.8% ^a^	10.1% ^a^		53.4% ^a^	8.5% ^a^		57.7% ^a^	11.4% ^a^	
65–69	58.0% ^b^	12.6% ^b^		56.6% ^b^	10.7% ^b^		59.2% ^b^	14.2% ^b^	
70–74	60.5% ^c^	15.8% ^c^		58.9% ^c^	13.8% ^c^		61.9% ^c^	17.5% ^c^	
75–79	60.3% ^c^	20.4% ^d^		59.6% ^c^	18.0% ^d^		60.9% ^c,d^	22.2% ^d^	
80–84	58.8% ^b^	25.4% ^e^		58.1% ^b,c^	23.0% ^e^		59.3% ^a,b,d^	26.9% ^e^	
≥85	54.3% ^a^	33.2% ^f^		55.6% ^a,b^	29.8% ^f^		53.5% ^e^	35.3% ^f^	
Urban or rural area			<0.001			<0.001			<0.001
Urban	56.7%	13.9%		54.5%	11.8%		58.5%	15.5%	
Rural	59.8%	18.7%		59.4%	16.2%		60.1%	20.7%	
Education			<0.001			<0.001			<0.001
Illiterate	59.8% ^a^	22.2% ^a^		60.6% ^a^	19.7% ^a^		59.6% ^a^	22.8% ^a^	
Primary school	58.6% ^b^	15.2% ^b^		57.5% ^b^	14.9% ^b^		59.7% ^a^	15.4% ^b^	
Junior secondary school	56.1% ^c^	11.5% ^c^		55.1% ^c^	11.3% ^c^		57.6% ^b^	11.8% ^c^	
Senior secondary school	55.3% ^c^	10.4% ^d^		53.5% ^c,d^	10.0% ^d^		58.2% ^a,b^	11.1% ^c^	
Tertiary	52.4% ^d^	9.2% ^d^		51.9% ^d^	7.6% ^e^		53.7% ^b^	13.5% ^b,c^	
University or above	57.6% ^a,b,c^	9.6% ^c,d^		56.3% ^b,c,d^	9.6% ^c,d,e^		61.0% ^a,b^	9.6% ^c^	
Marital status			<0.001			<0.001			<0.001
Married	56.9% ^a^	13.0% ^a^		55.3% ^a^	12.2% ^a^		58.6% ^a^	13.9% ^a^	
Widowed	60.5% ^b^	23.6% ^b^		61.2% ^b^	21.4% ^b^		60.3% ^b^	24.3% ^b^	
Divorced	64.4% ^b,c^	14.3% ^a,c^		66.1% ^b,c^	13.0% ^a,c^		62.0% ^a,b^	16.2% ^a^	
Unmarried	69.2% ^c^	18.3% ^c^		70.1% ^c^	17.6% ^c^		57.4% ^a,b^	27.8% ^b^	
Ethnicity			<0.001			0.112			0.001
Han	57.9%	15.9%		56.4%	13.6%		59.1%	17.7%	
Non-Han	61.7%	17.9%		61.1%	15.5%		62.2%	19.8%	
Living status			<0.001			<0.001			<0.001
Living alone	66.7%	24.4%		68.0%	20.7%		66.0%	26.2%	
Not living alone	56.7%	14.7%		55.3%	12.9%		57.9%	16.2%	
Health checkup within 1 year			<0.001			<0.001			<0.001
No	57.5%	18.1%		56.3%	15.4%		58.3%	20.1%	
Yes	58.5%	14.8%		56.8%	12.8%		59.8%	16.5%	
Hospitalized within 1 year			<0.001			<0.001			<0.001
No	57.7%	11.7%		55.7%	9.7%		59.3%	13.4%	
Yes	58.9%	25.6%		58.8%	23.0%		59.1%	27.5%	
Economic status			<0.001			<0.001			<0.001
Very rich	46.4% ^a^	10.0% ^a^		45.3% ^a^	9.3% ^a,b^		47.2% ^a^	10.5% ^a^	
Rich	53.4% ^b^	10.5% ^a^		51.2% ^b^	8.5% ^b^		55.3% ^b^	12.1% ^a^	
Adequate	58.3% ^c^	14.3% ^b^		56.4% ^c^	12.2% ^a^		59.8% ^c^	15.9% ^b^	
Poor	61.8% ^d^	22.4% ^c^		62.0% ^d^	19.3% ^c^		61.6% ^d^	24.8% ^c^	
Very poor	58.0% ^c^	33.2% ^d^		59.1% ^c^	31.3% ^d^		57.2% ^b^	34.7% ^d^	
Medicare			0.310			0.140			0.523
Yes	58.1%	16.0%		56.6%	13.7%		59.3%	17.8%	
No	57.8%	18.0%		59.5%	15.9%		56.5%	19.8%	
Convenience of medical cost reimbursement			<0.001			<0.001			<0.001
Highly convenient	57.4% ^a^	14.9% ^a^		55.4% ^a^	12.5% ^a^		59.0% ^a^	16.8% ^a^	
Convenient	57.8% ^a^	16.0% ^b^		56.2% ^a^	13.8% ^b^		59.0% ^a^	17.7% ^b^	
Less convenient	59.6% ^b^	16.6% ^b^		59.1% ^b^	14.1% ^b^		60.1% ^a^	18.6% ^b^	
Inconvenient	60.4% ^b^	21.1% ^c^		59.2% ^b^	19.4% ^c^		61.4% ^a^	22.4% ^c^	
Highly inconvenient	59.2% ^a,b^	21.6% ^c^		60.8% ^b^	18.0% ^c^		57.9% ^a^	24.6% ^c^	
Comorbidities			<0.001			<0.001			<0.001
<1	26.7%	1.6%		24.5%	1.2%		28.9%	1.9%	
≥1	66.1%	19.7%		66.3%	17.5%		66.0%	21.4%	
ADL disability			<0.001			<0.001			<0.001
Yes	19.5%	80.1%		20.1%	79.2%		19.1%	80.7%	
No	60.4%	12.1%		58.6%	10.2%		61.9%	13.7%	

ADL, activities of daily living.

The superscript letters a, b, c, d, e, and f indicate the difference in the prevalence of pre-frailty and frailty among different groups (adjusted *p*-values).

### Prevalence of frailty and pre-frailty in elderly hypertensive patients in different regions of China

**FIGURE 3:**
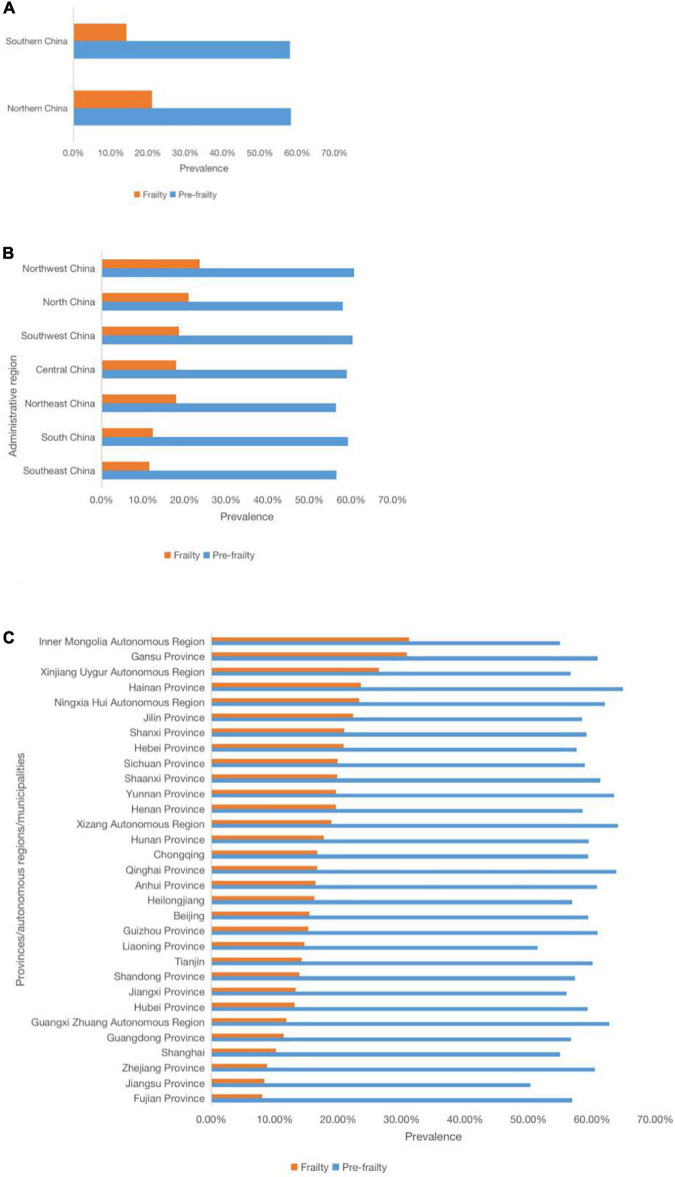
Prevalence of frailty and pre-frailty in older adults with hypertension in different regions of mainland China. **(A)** Prevalence of frailty and pre-frailty in older adults with hypertension in different provinces, municipalities, and autonomous regions. **(B)** Prevalence of frailty and pre-frailty in older adults with hypertension in different administrative regions. **(C)** Prevalence of frailty and pre-frailty in older adults with hypertension in different Northern and Southern China.

Prevalence of frailty (χ^2^ = 1637.632, *P* < 0.001) and prevalence of pre-frailty (χ^2^ = 322.241, *P* < 0.001) of hypertensive elderly in different regions were significantly different (Figure 3 and Supplementary Table 1). The highest prevalence of frailty among elderly with hypertension in 31 provinces, municipalities, and autonomous regions was in Inner Mongolia Autonomous Region (31.2%) and the lowest was in Fujian Province (8.0%), with the former being 3.9 times higher than the latter, and the highest prevalence of pre-frailty was in Hainan Province (64.9%) and the lowest was in Jiangsu Province (50.3%), with the former being 1.3 times higher than the latter. The prevalence of frailty in different administrative regions was different (χ^2^ = 1010.984, *P* < 0.001). prevalence of frailty was highest in Northwest China (23.6%), followed by North China (20.9%), Southwest China (18.6%); Northeast China, and Central China were in the middle (both 18.0%), while South China was lower (12.3%), with lowest in Southeast China (11.5%). The prevalence of pre-frailty in different administrative regions was different (χ^2^ = 79.284, *P* < 0.001). The prevalence of frailty of hypertensive elderly in northern China was higher than that in southern China (21.0% vs. 14.2%, χ^2^ = 508.215, *P* < 0.001), while the prevalence of pre-frailty of hypertensive elderly in northern China was not different from that in southern China (58.3% vs. 58.0%, χ^2^ = 0.530, *P* = 0.467).

### Multinomial logistic regression analysis of associated with frailty and pre-frailty in elderly hypertension

Results of multinomial logistic regression see Table 3. Poor economic status, living alone, ADL disability, and comorbidities are highly correlated factors of frailty and pre-frailty in hypertensive elderly.

**TABLE 3:** Related factors associated with frailty and pre-frailty by multinomial logistic regression of older adults with hypertension.

Variable		Pre-frailty vs. robust				Frailty vs. robust			
		**OR**	**95%CI**		***P*-value**	**OR**	**95%CI**		***P*-value**
			**Lower**	**Upper**			**Lower**	**Upper**	
Sex	Male	1 (ref)							
	Female	1.135	1.086	1.185	0.001	1.205	1.130	1.286	<0.001
Age (years)	60–64	1 (ref)							
	65–69	1.161	1.100	1.226	<0.001	1.322	1.215	1.437	<0.001
	70–74	1.423	1.339	1.513	<0.001	1.805	1.650	1.975	<0.001
	75–79	1.750	1.632	1.878	<0.001	2.531	2.294	2.793	<0.001
	80–84	2.229	2.040	2.436	<0.001	3.627	3.224	4.080	<0.001
	≥85	2.931	2.582	3.327	<0.001	5.795	4.951	6.784	<0.001
Urban or rural area	Urban	1 (ref)							
	Rural	1.270	1.214	1.328	<0.001	1.491	1.400	1.588	<0.001
Marriage	Married	1 (ref)							
	Widowed	1.065	1.000	1.133	0.049	1.118	1.028	1.216	0.009
	Divorced	1.087	0.841	1.404	0.525	1.131	0.794	1.612	0.496
	Unmarried	1.656	1.261	2.175	<0.001	1.503	1.069	2.115	0.019
Education	Illiterate	1 (ref)							
	Primary school	0.823	0.778	0.870	<0.001	0.734	0.681	0.791	<0.001
	Junior high school	0.716	0.670	0.766	<0.001	0.564	0.512	0.622	<0.001
	Senior high school	0.688	0.631	0.751	<0.001	0.507	0.444	0.579	<0.001
	Tertiary education	0.605	0.529	0.691	<0.001	0.444	0.355	0.554	<0.001
	University or higher	0.620	0.522	0.737	<0.001	0.445	0.336	0.589	<0.001
Ethnicity	Han	1 (ref)							
	Other	1.146	1.037	1.265	0.007	0.962	0.840	1.101	0.573
Living alone	No	1 (ref)							
	Yes	5.554	5.058	6.098	<0.001	9.701	8.670	10.855	<0.001
Medical checkup within the previous year	Yes	1 (ref)							
	No	1.062	1.017	1.109	0.007	1.158	1.090	1.231	<0.001
Hospitalized within the previous year	No	1 (ref)							
	Yes	1.553	1.479	1.631	<0.001	3.082	2.893	3.284	<0.001
Region	South	1 (ref)							
	North	1.373	1.307	1.443	<0.001	1.924	1.801	2.056	<0.001
Medicare	Yes	1 (ref)							
	No	0.849	0.676	1.065	0.156	0.892	0.657	1.212	0.464
Economic status	Very rich	1 (ref)							
	Rich	1.608	1.368	1.890	<0.001	1.504	1.132	1.997	0.005
	Adequate	2.484	2.123	2.906	<0.001	3.194	2.424	4.207	<0.001
	Poor	5.221	4.427	6.159	<0.001	10.764	8.119	14.270	<0.001
	Very poor	10.489	8.450	13.020	<0.001	38.039	27.607	52.412	<0.001
Medical reimbursement	Very convenient	1 (ref)							
	Convenient	1.040	0.992	1.090	0.102	1.064	0.995	1.138	0.071
	Less convenient	1.132	1.064	1.205	<0.001	1.117	1.024	1.219	0.013
	Inconvenient	1.412	1.248	1.597	<0.001	1.732	1.478	2.029	<0.001
	Very inconvenient	1.399	1.174	1.667	<0.001	1.714	1.371	2.144	<0.001
Comorbidities	<1	1 (ref)							
	≥1	16.972	16.151	17.835	<0.001	149.581	128.120	174.636	<0.001
ADL disability	No	1 (ref)							
	Yes	40.557	24.731	66.510	<0.001	893.019	542.669	1469.558	<0.001

CI, confidence interval; OR, odds ratio; ADL, activities of daily living.

## Discussion

Our study showed a self-reported prevalence of hypertension in the elderly was 36.9%, consistent with a national, stratified, multi-stage random sampling cross-sectional survey of hypertension from 2012 to 2015, the data shows that the prevalence of people aged 60 or above is 53.2%, with an awareness rate is 57.1% (1).

The current cross-sectional study had a larger sample size than previous studies of frailty in older adults with hypertension in China, covering every province, municipality, and autonomous region in mainland China, using a rigorous sampling design and standard methods to ensure survey quality. Our study accurately reported an age-sex standardized prevalence of frailty and pre-frailty in Chinese elderly with hypertension of 16.1% (95% CI 15.8–16.3%) and 58.1% (95% CI 57.7–58.4%), respectively, and estimated the number of frail and pre-frail elderly hypertensives in mainland China in 2020 to be 22.5 (22.2–22.9) million and 81.5 (81.0–82.0) million. Our study reported a higher prevalence of frailty in elderly hypertensive patients than a previous study, Ma et al. reported a 13.8% prevalence of frailty in a national study that included 3,844 elderly hypertensive patients in a comprehensive assessment of the elderly in China (15). Our findings and those of Ma et al. suggest that frailty is prevalent among older hypertensive patients in China. However, we report a lower prevalence compared to foreign countries, with a Korean study reporting a 48.4% prevalence of frailty in older hypertensive patients in the community (20), the systolic pressure intervention trial (SPRINT) showed an overall prevalence of 27.6% for frailty, with FI > 0.21 as the threshold for the diagnosis of frailty (21). Our study and the above studies suggest that hypertension is an important risk factor for frailty and that hypertension-related cardiovascular complications promote the development of frailty.

Our large nationally representative sample study found that frail older hypertensive patients were older and had higher rates of hospitalization in the past year, ADL disability and comorbid chronic diseases compared to non-frail older hypertensive patients. These characteristics suggest that frail older hypertensive patients are prone to adverse events. In a longitudinal aging study, Ma et al. documented frailty in 1,111 hypertensive elderly in China aged 60 years or above and found that after 8 years of follow-up, compared with hypertensive patients without frailty, hypertensive patients with frailty had higher mortality (22). A study by Ravindrarajah et al. included 140,000 older adults over 80 years who had complete electronic health records, these researchers’ analysis showed that, regardless of whether patients received antihypertensive treatment, the mortality of patients increased with their severity of frailty (23). A recent meta-analysis showed that frail elderly hypertension patients are prone to accidents (10). In short, older hypertensive patients who are frail have a higher risk of adverse events than older hypertensive patients who are not frail.

These characteristics in our study showed that frail older hypertensive patients represent a complex and highly vulnerable group of people. Whether frail older hypertensive patients benefit from antihypertensive therapy has received much attention because frail older hypertensive patients are often excluded from clinical trials of antihypertensive therapy, and few trials have studied antihypertensive therapy in frail hypertensive patients. Hypertension in the very elderly trial (HYVET) *post hoc* analysis showed that frail older individuals could benefit from antihypertensive treatment compared with placebo (24), and the SPRINT subgroup study showed that older adults ≥ 75 years of age could benefit from intensive antihypertensive therapy compared with standard treatment, regardless of whether they were frail, albeit marginally significant (*P* = 0.06). However, in the SPRINT study, the intensive antihypertensive group had a higher risk of adverse events, for example, hypotension, syncope, and electrolyte disturbance (21). Rea et al. reported that adherence to antihypertensive medications in Italian older adults was associated with total and cardiovascular mortality (25). Although this study was conducted indirectly, medication adherence can help better control blood pressure (26, 27). These studies suggested that frail older hypertensive patients may benefit from antihypertensive or intensive treatment, although they are prone to treatment-related complications. However, due to the relative health of selected study participants and inconsistent evaluation methods, many studies excluded individuals who were unfit to participate in the survey. Some studies have found that frailty is one of the critical factors affecting the benefits of antihypertensive treatment in older adults (10, 28). Another study showed that frailty makes older people who take blood pressure lowering drugs more susceptible to injury (12). A meta-analysis showed that systolic blood pressure greater than or less than 140 mmHg among frail people did not affect mortality, ordinary people with systolic blood pressure less than 140 mmHg are less likely to die (29). According to a newly published longitudinal study, those with poorly controlled blood pressure had a higher risk of becoming frail (HR = 1.96, 95% CI 1.49–2.56, *p*0.001), and their frailty ratings were positively correlated with them (β = 0.015; 95% CI 0.011–0.019; *p*0.001) (30). The 2019 Chinese Guideline for Hypertension in Older Persons, the European Society of Hypertension, the European Geriatric Medicine Society, and other organizations strongly underlined that frailty assessment is a crucial component of the proper therapy of hypertension in older adults. One of the essential prerequisites before treatment begins is that the degree of frailty should be carefully assessed to determine those patients who are unlikely to tolerate antihypertensive therapy and those that will benefit from the antihypertensive treatment and to promptly identify changes in the frailty state in case their treatment needs to be adjusted (13, 14).

The results of our study supported earlier research on frailty reason (15, 31–33). Frailty and pre-frailty were more prevalent among women, rural residents, widowed/unmarried, those living alone, those with inconvenient medical reimbursement, those with financial hardship, those with ADL disabilities, and those with comorbid chronic disease. Older hypertensive patients with low education, who belonged to an ethnic minority, had been hospitalized in the previous year, and had not received physical examination in the previous year had a higher prevalence of frailty.

Our study found that compared to older men with hypertension, older women with hypertension were relatively older, had higher rates of widowhood and living alone, had lower rates of economic affluence, good education and had a medical examination in the past year, and had higher rates of comorbid chronic disease and ADL disability. These differences in demographic characteristics might partially explain the sex differences in frailty in older hypertensive patients. Studies showed that the prevalence of frailty increases with age, which is associated with age-related organ degeneration and reduced reserve capacity, and age is considered an independent risk factor for frailty (7, 34). Widowhood and living alone are predisposing factors for loneliness and depression in older people and are risk factors for frailty in older people (35), suggesting the need for enhanced social support for older people with hypertension who are widowed and living alone. Good education is associated with health behaviors and economic status, and good economic status facilitates access to better healthcare, which are protective factors in reducing the incidence of frailty in older people (36, 37). Medical examination helps to detect early risk factors for frailty. Chronic disease and ADL disability and frailty are mutually reinforcing. Management of risk factors for frailty in older hypertensive patients can help prevent frailty. A combination of interventions such as management of comorbid chronic diseases, exercise, and nutritional interventions can help reverse or slow the progression of frailty in elderly hypertensive patients with mild to moderate frailty, and reduce the risk of disability in older people with hypertension. These findings could help the government develop policies to prevent and improve the frailty of older women with hypertension.

We found that the frailty prevalence of older hypertensive patients in the economically underdeveloped western and northern regions was relatively high, while the economically developed coastal areas were relatively low. The 2012–2015 Chinese hypertension stratified multi-stage random sampling cross-sectional survey data showed that hypertension awareness rate, treatment rate, and control rate in rural older adults were significantly lower than in urban (1). Therefore, more medical assistance and services are needed in rural areas and less economically developed western and northern regions to improve the physical quality of older adults and to reduce the likelihood of frailty in older adults with hypertension (15).

With the 2020 China Census showing that China already has the largest elderly population in the world and is rapidly entering deep aging, the burden of aging in China is set to increase further (38). The health of older people is the most prominent issue in an aging society, and over the past three decades China has completed an epidemiological transition from communicable to non-communicable diseases (39). There are currently about 140 million elderly people with hypertension in China, making it the most prevalent chronic non-communicable disease among the elderly in China. Hypertension is the most important and modifiable risk factor for cardiovascular disease mortality in the elderly population in both urban and rural areas (13). Our study accurately estimates the frailty burden of hypertension in older adults in China, but current evidence suggests that frailty cannot be overcome by addressing traditional chronic conditions alone. Studies have shown that frail older adults with hypertension have increased vulnerability to stress, increased risk of disability, functional decline, hospitalization and death, and can lead to increased demand for long-term care and healthcare costs for older adults with hypertension (40). The management of hypertension in the elderly therefore needs to move from a disease-centered model to a patient-centered model, and a new concept and approach to the treatment and management of the frailty of hypertension in the elderly must be adopted. Priority is given to primary prevention strategies, firstly to raise awareness of frailty in elderly hypertensive patients through systematic health education; and secondly to prevent frailty through risk factor interventions in elderly hypertensive patients with modifiable risk factors for frailty and pre-frailty. For elderly hypertensive patients with frailty and pre-frailty, comprehensive interventions such as exercise, nutritional interventions and co-morbidity management are used to reverse frailty or delay the progression of frailty and reduce the occurrence of negative clinical events. Our study also found sex, rural-urban and geographical differences in the frailty of hypertension in older people, and public health strategies should be targeted to enhance the prevention of frailty in older people with hypertension in women, rural, northern and western regions, and to promote healthy aging in older people with hypertension.

## Limitations

Our study has several limitations. First, the diagnosis of hypertension was self-reported and memory errors might occurred. Secondly, there was no information on hypertension-related variables such as disease duration, blood pressure class, target organ status, antihypertensive medication, and whether blood pressure reached the target with antihypertensive medication, which may be relevant to the frail progression of older people with hypertension. Thirdly, information on the severity of comorbidities and the type of pathology. Defining comorbidities in terms of severity, and particularly type of disease, would better characterize the population. Fourth, the frailty assessment lacked items on cognitive function and instrumental activities of daily living items, which are important elements in the assessment of geriatric syndromes. Fifth, this study is cross-sectional and therefore cannot determine the causal relationship between the factors of interest and frailty. Therefore, we will keep exploring more.

## Conclusion

We suggest that frailty assessment be a standard approach in managing older hypertensive patients. Controlling the risk factors of frailty, strengthening the prevention of frailty, improving the management of frailty, and raising public awareness of frailty will effectively reduce the burden of older hypertensive patients in China.

## Data availability statement

The original contributions presented in this study are included in the article/Supplementary material, further inquiries can be directed to the corresponding author.

## Ethics statement

The study protocol was approved by the National Bureau of Statistics (No. [2014] 87) and the Ethics Committee of Beijing Hospital (2021BJYYEC-294-01). The patients/participants provided their written informed consent to participate in this study.

## Author contributions

D-PL, Q-XZ, JL, and X-ZZ conceived and designed the study. D-PL, JL, and Q-XZ obtained funding. X-ZZ and L-BM analyzed the data. X-ZZ and NJ drafted the manuscript. JS, NJ, XQ, J-BH, XH, HL, H-XX, Y-YL, and HW helped to interpret the results. D-PL was the guarantor and contributed to the critical revision of the manuscript for important intellectual content. All authors reviewed and approved the final manuscript.
