# Supplementary figures and images for: A study on the prevalence and related factors of frailty and pre-frailty in the older population with hypertension in China: A national cross-sectional study
Source: Front Cardiovasc Med. 2023 Jan 12;9:1057361. doi: 10.3389/fcvm.2022.1057361 (PMC9877294; doi:10.3389/fcvm.2022.1057361)

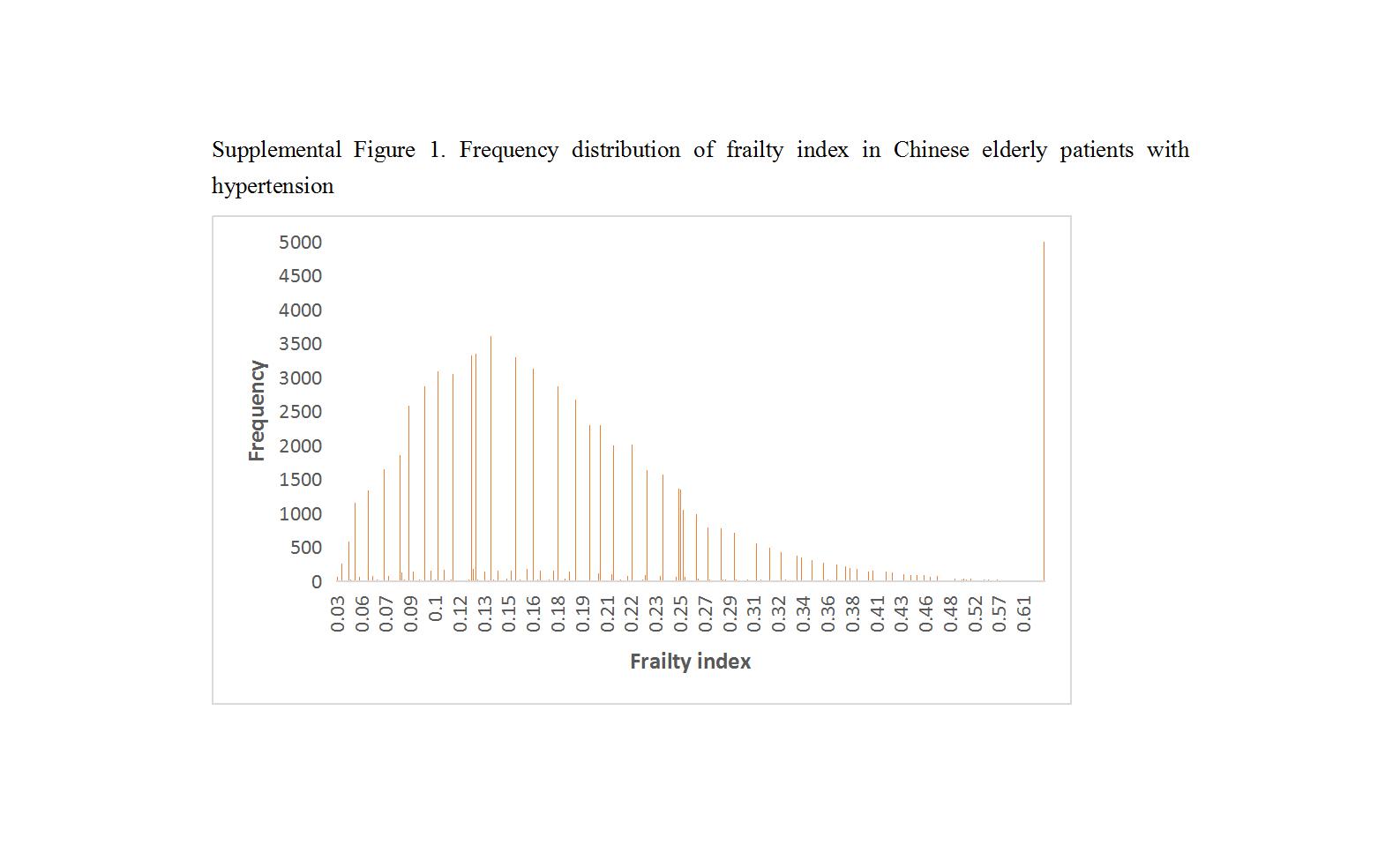

Supplement: Supplementary file 1 [file Image_1.JPEG]
