# Supplementary material for: A study on the prevalence and related factors of frailty and pre-frailty in the older population with hypertension in China: A national cross-sectional study
Source: Front Cardiovasc Med. 2023 Jan 12;9:1057361. doi: 10.3389/fcvm.2022.1057361 (PMC9877294; doi:10.3389/fcvm.2022.1057361)
Supplement: Supplementary file 2 [file Table_1.docx]

Supplemental Table 1. Prevalence of frailty and pre-frailty in older adults with hypertension in 31 provinces/autonomous regions/municipalities and administrative Regions in mainland China in 2015

| Province/autonomous region/municipality | Prevalence of pre-frailty | Prevalence of frailty |
| --- | --- | --- |
| Fujian Province | 56.9% | 8.0% |
| Jiangsu Province | 50.3% | 8.3% |
| Zhejiang Province | 60.4% | 8.8% |
| Shanghai | 55.0% | 10.2% |
| Guangdong Province | 56.7% | 11.4% |
| Guangxi Zhuang Autonomous Region | 62.7% | 11.8% |
| Hubei Province | 59.3% | 13.1% |
| Jiangxi Province | 56.0% | 13.3% |
| Shandong Province | 57.3% | 13.9% |
| Tianjin | 60.1% | 14.2% |
| Liaoning Province | 51.4% | 14.7% |
| Guizhou Province | 60.9% | 15.3% |
| Beijing | 59.4% | 15.5% |
| Heilongjiang Province | 56.9% | 16.2% |
| Anhui Province | 60.8% | 16.4% |
| Qinghai Province | 63.8% | 16.7% |
| Chongqing | 59.4% | 16.7% |
| Hunan Province | 59.5% | 17.7% |
| Tibet Autonomous Region | 64.1% | 18.9% |
| Henan Province | 58.5% | 19.6% |
| Yunnan Province | 63.5% | 19.6% |
| Shaanxi Province | 61.3% | 19.8% |
| Sichuan Province | 58.9% | 19.9% |
| Hebei Province | 57.6% | 20.8% |
| Shanxi Province | 59.1% | 20.9% |
| Jilin Province | 58.4% | 22.3% |
| Ningxia Hui Autonomous Region | 62.0% | 23.3% |
| Hainan Province | 64.9% | 23.5% |
| Xinjiang Uygur Autonomous Region | 56.6% | 26.4% |
| Gansu Province | 60.9% | 30.8% |
| Inner Mongolia Autonomous Region | 55.0% | 31.2% |
| Administrative Regions |  |  |
| Northwest China | 60.7% | 23.6% |
| Northeast China | 56.4% | 18.0% |
| North China | 58.0% | 20.9% |
| Central China | 59.0% | 18.0% |
| South China | 59.2% | 12.3% |
| Southwest China | 60.3% | 18.6% |
| Southeast China | 56.5% | 11.5% |
